# A New Insight in Determining the Percolation Threshold of Electrical Conductivity for Extrinsically Conducting Polymer Composites through Different Sigmoidal Models

**DOI:** 10.3390/polym9100527

**Published:** 2017-10-19

**Authors:** Mostafizur Rahaman, Ali Aldalbahi, Periyasami Govindasami, Noorunnisa P. Khanam, Subhendu Bhandari, Peter Feng, Tariq Altalhi

**Affiliations:** 1 Department of Chemistry, College of Science, King Saud University, Riyadh 11451, Saudi Arabia; pkandhan@ksu.edu.sa; 2 Department of Mechanical and Industrial Engineering, Qatar University, Doha 2713, Qatar; noor.pathan@qu.edu.qa; 3 Department of Plastics and Polymer Engineering, Maharashtra Institute of Technology, Aurangabad, Maharashtra 431010, India; subhenduonly@gmail.com; 4 Department of Physics, University of Puerto Rico, San Juan, PR 00936-8377, USA; peter.feng@upr.edu; 5 Department of Chemistry, Faculty of Science, Taif University, Taif 21974, Saudi Arabia; Ta.altalhi@tu.edu.sa

**Keywords:** polymer composites, electrical conductivity, percolation threshold, sigmoidal models

## Abstract

The electrical conductivity of extrinsically conducting polymer composite systems passes through a transition state known as percolation threshold. A discussion has been made on how different Sigmoidal models (S-models), such as Sigmoidal–Boltzmann (SB), Sigmoidal–Dose Response (SD), Sigmoidal–Hill (SH), Sigmoidal–Logistic (SL), and Sigmoidal–Logistic-1 (SL-1), can be applied to predict the percolation threshold of electrical conductivity for ethylene vinyl acetate copolymer (EVA) and acrylonitrile butadiene copolymer (NBR) conducting composite systems filled with different carbon fillers. An interesting finding that comes from these observations is that the percolation threshold for electrical conductivity determined by SB and SD models are similar, whereas, the other models give different result when estimated for a particular composite system. This similarity and discrepancy in the results of percolation threshold have been discussed by considering the strength, weakness, and limitation of the models. The percolation threshold value for the composites has also been determined using the classical percolation theory and compared with the sigmoidal models. Moreover, to check the universal applicability, these Sigmoidal models have also been tested on results from some published literature. Finally, it is revealed that, except SL-1 model, the remaining models can successfully be used to determine the percolation threshold of electrical conductivity for extrinsically conductive polymer composites.

## 1. Introduction

Most of the polymers are inherently insulating in nature. However, these insulating polymers can be made semi-conducting/conducting by the inclusion of a certain amount of conducting fillers. Actually, these polymer/filler systems are known as extrinsically conductive polymer composites. Generally, different forms of filler, such as particulate, flake, tubular or fibrous fillers, are used as inclusion in the host polymer matrix. The use of particulate carbon black, carbon nanotubes, carbon fibers, and metallic particles as conducting fillers has been reported by several authors [1,2,3,4,5]. The quantity of filler at which an extrinsically conducting polymer composite system undergoes transition from its insulating to conducting state is known as percolation threshold [2]. Electrical percolation of extrinsically conductive composites depends on filler type, shape and size; filler dispersion and distribution in the composite; and the processing condition of the composite systems [3,6]. At percolation threshold, the conductive components are dispersed in such a manner that one or more continuous conductive networks are formed throughout the polymer matrix.

Several Models and equations have been proposed to explain the percolation threshold of polymer conductive composites. Kirkpatrick proposed the behavior of electrical conductivity above percolation threshold, which follows the power law relationship [7]. Many studies have shown that the theory works well with the polymer composites filled with carbon nanotubes [8], carbon blacks [9], metal particles [10], and intrinsically conducting polymers in insulating polymer matrix [11]. Malliaris and Turner formalized the theoretical percolation model based on the initial formation of infinite long chain of metallic powder in the polymer matrix, where the conductive particles uniformly cover the surface of large polymer particles [5]. A percolation model has been developed by Janzen based on the concept of mean number of contacts between the filler particles [12]. Other than these models, many approaches have been made to explain the percolation behavior of conducting polymer composite systems [13,14,15,16,17,18]. However, no such model alone predicts the percolation threshold of electrical conductivity when one considers all of the experimental results of conductivity. However, the prediction of percolation threshold through different S-model is scant.

In our present study, we have investigated the percolation threshold of electrical conductivity by applying different Sigmoidal models such as Sigmoidal–Boltzmann (SB), Sigmoidal–Dose response (SD), Sigmoidal–Hill (SH), Sigmoidal–Logistic (SL), and Sigmoidal–Logistic-1 (SL-1). A mathematical approach has been made to determine the percolation threshold through all these models. These models will produce the percolation threshold based only on the experimental results. The obtained results of percolation threshold from these models have also been compared with the result of percolation threshold calculated from classical percolation theory. Moreover, their applicability to determine the percolation threshold value has also been tested with the conductivity data of some published literature.

## 2. Materials, Methods, and Characterization

### 2.1. Materials

The base polymer matrices, Acrylonitrile Butadiene Rubber (NBR, mooney viscosity, ML _1+4_ at 100 °C is 45) with acrylonitrile content of 33%, was supplied by Japan Synthetic Rubber Co., Ltd. (Tokyo, Japan) and Ethylene vinyl acetate rubber (EVA-2806, mooney viscosity, ML _1+4_ at 100 °C is 20) with vinyl acetate content 28% (MFI = 6) was purchased from NOCIL, Mumbai, India. The conductive fillers, conductex (SC Ultra bead) carbon black (CCB), supplied by Columbian Chemicals Company, Atlanta, GA, USA; printex XE2 carbon black (PCB), procured from Degussa Canada Limited, Burlington, ON, Canada; and conductive short carbon fiber (SCF) (RK 30/12), obtained from RK Carbon Fiber, Leatherhead, UK, were used in the preparation of the composites. The physical characteristics of conductex and printex carbon blacks are reported in Table S1, whereas short carbon fiber has been reported in Table S2 and some discussion of their physical characteristics are made in the Supplementary Materials. 

Curing agent, Dicumile peroxide (DCP), MP = 80 °C and a purity of 98%, was supplied by Aldrich chemical company, St. Louis, MO, USA. Tri Allyl Cyanurate (TAC), supplied by E. Merck (India limited), Mumbai, India, was used as *co*-vulcanizing agent. Antioxidant, 1,2-Dihydro-2,2,4-trimethylquinoline (TQ, polymerized), was obtained from Lanxess (India) Private Ltd. (Bharuch, India). 

### 2.2. Preparation of Composites and Samples

The composites of EVA and NBR were prepared with the help of a Brabender Plasticorder (PLE 330, Brabender GmbH & Co. KG, Duisburg, Germany) and two-roll mixing mill (Santec Exim Pvt Ltd., Manesar, India). Initially, the neat EVA and neat NBR was separately melted in a Brabender at 120 °C for 6 min at the shear rate 60 rpm to form a compact mass. The conductive additives (carbon blacks or carbon fiber) along with other ingredients (TAC, TQ, and DCP) were mixed with neat EVA and neat NBR separately in a two-roll mixing mill at room temperature in a sequential manner, as per the formulations given in Table 1. The additives and other ingredients were taken as per hundred parts of polymers. Accordingly, the volume ratios of polymers and additives were also calculated, as shown in Table 1. The calculation method of volume fraction of fillers is shown in Supplementary Materials (C1). The curing time for various composites was measured using Monsanto rheometer R-100S (Gomaplast Machinery, Inc., Wooster, OH, USA) at temperature 160 °C and time duration 1 h. The test specimens of different composites were prepared under compression molding at curing temperature 160 °C for the specific curing time.

Polymers, carbons, and composites designation: polymers NBR and EVA are designated as N and E, respectively; and the carbons CCB, PCB, and SCF are designated as C, P, and F, respectively. Accordingly, the composites of NBR and Conductex carbon black are designated as NC and so on.

### 2.3. Measurement of DC Resistivity

The direct-current (DC) volume resistivity of different composites vary over wide range of resistivity were measured by using two sets of instruments: Agilent 4339B (Santa Clara, CA, USA, High Resistance Meter attached with Agilent 16008B Resistivity Cell, Santa Clara, CA, USA) for high resistance, and GOM-802 (GW Instek DC milli Ohm Meter, Good Will Instrument Co., Ltd., New Taipei City, Taiwan) for low resistance measurements.

## 3. Results and Discussion

### 3.1. Origin of the Concept

**Figure 1:**
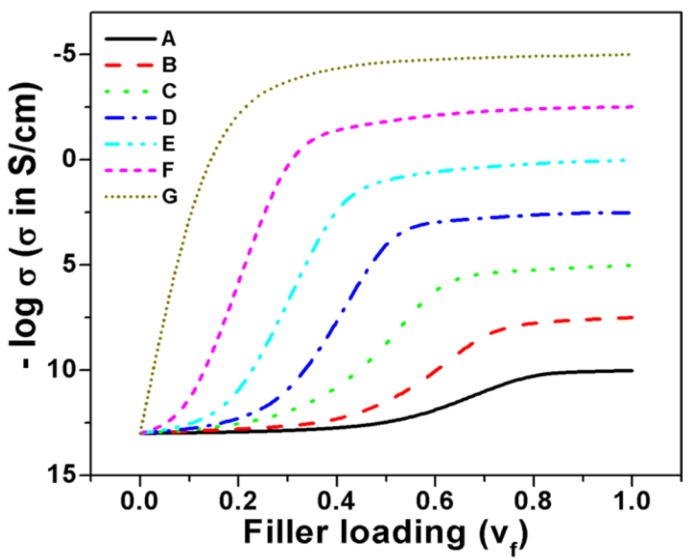
Variation of conductivity against filler loading (volume fraction) for polymer composite systems, (A–F) S-shaped curve, (G) non S-shaped curve.

**Figure 2:**
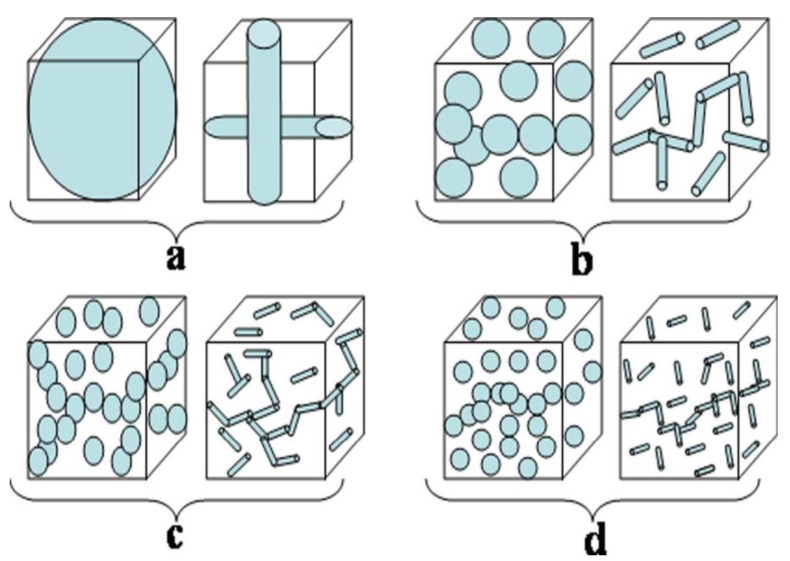
Dimension of particulate and fibrous fillers inside a polymer composite, dimension of filler in (**a**) is greater and (**b**–**d**) is smaller than the sample dimension.

**Figure 3:**
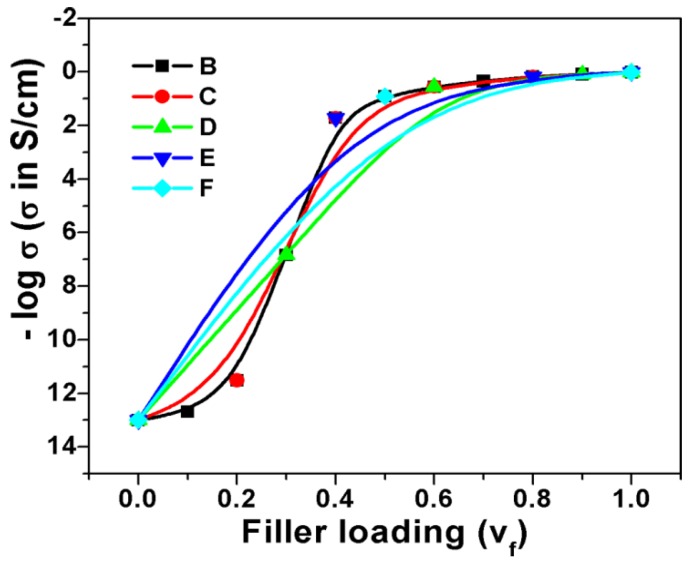
Variation of conductivity against filler loading (volume fraction) for same polymer composite at different interval of volume fraction, point of interval is decreasing from B to F.

In many cases, the progression of a dependent parameter starts with very small beginning that accelerates and approaches a climax with respect to the independent parameter. If represented graphically, this type of curve is called Sigmoidal curve or simply S-shaped curve. The variation of electrical conductivity with respect to conductive filler loading in insulation polymer matrix also follows the same trend: initially, the increase in conductivity starts with small beginning at low filler loading followed by sharp increase in percolation region and then finally becomes asymptotic in nature with further addition of conductive filler. This similarity of electrical conductivity curve (against conductive additive loading) with S-shaped curve motivated us to make study on this field to determine the electrical percolation threshold of extrinsically conductive composite systems. To proceed further on this study, the variation of dependent parameters such as conductivity with respect to volume fraction of filler for extrinsically conducting composite systems can be graphically represented (Figure 1). It is observed in Figure 1 that all curves are S-shaped, except G. However, the nature of the S-shaped curves depends on the shape, size, aspect ratio, dispersion, distribution, structure, and conductivity of the filler particles. The shape, size, and/or aspect ratio of carbon fillers and their composites are shown in Figure S1 (see Supplementary Materials, Morphology Section of Carbons and their Composites). The percolation threshold for all the composite systems that exhibit S-shaped nature can be obtained through Sigmoidal model. Actually, the filler dimension inside the composite also plays an important role in controlling the S-shaped behavior of the curves. If the dimension (length or diameter) of filler (fibrous or particulate) is greater than the dimension of the sample (shown in Figure 2a), then the chances of finding S-shaped curve is nil, whereas, for the other cases (illustrated in Figure 2b–d), the S-shaped curved can be obtained. Moreover, the intervals of volume fraction of filler in the polymer composite also determine the chances of obtaining and nature of S-shaped curve. A large interval of volume fraction of filler leads many times to devoid from the S-shaped nature of curves, as shown in Figure 3. 

### 3.2. Derivation of Percolation Threshold and Sigmoidal–Boltzmann Model

The mathematics behind the determination of percolation threshold for all the Sigmoidal models presented herein is quite simple and similar. For the sake of simplicity, the equation based on Sigmoidal–Boltzmann model that been considered herein can be given as:

(1) $$Y=A_{2}+\frac{A_{1}-A_{2}}{1+e^{\left(\frac{x-x_{0}}{\Delta x}\right)}}$$

**Figure 4:**
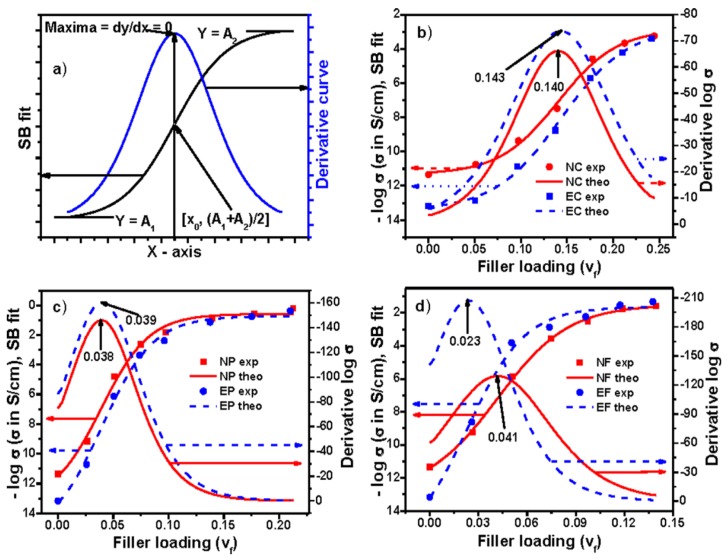
Experimental, theoretical, and derivative curves: (**a**) an ideal curve; (**b**) Conductex black filled; (**c**) Printex black filled; and (**d**) SCF filled composites based on Sigmoidal–Boltzmann model.

On differentiation of the above equation, we have:

(2) $$\frac{dy}{dx}=\frac{(A_{2}-A_{1})e^{\frac{x-x_{0}}{\Delta x}}}{\Delta x{\left(1+e^{\frac{x-x_{0}}{\Delta x}}\right)}^{2}}$$

where *Y* is the dependent parameter of electrical conductivity at the corresponding independent parameter that is the volume fraction of filler *x*. *A*_1_ and *A*_2_ are the initial and final values of electrical conductivity. ∆*x* is the slope which indicates the steepness of the curve and *x*_0_ is the *x*-axis value at the corresponding *y*-axis value equal to (*Y*_1_ + *Y*_2_)/2. An ideal curve based on Equation (1) has been given in Figure 4a, which exhibits S-shaped behavior. The nature of this curve is found to be almost similar to the variation of electrical conductivity with respect to the volume fraction of filler for the present composite systems. Percolation threshold is the phenomenon where rate of change of any electrical properties exhibits maximum. Actually, the first order differentiation of Equation (1) (i.e., Equation (2)) will produce a maximum (shown in Figure 4a) and the magnitude of this maximum can be obtained by equating the second order differentiation of Equation (1) to zero. This results *x* = *x*_0_. Thus, in Equation (1), *x*_0_ indicates the value of percolation threshold. Similarly, the point where the percolation threshold for the other Sigmoidal models exists can also be derived by applying the same methodology. The percolation thresholds for these models have been presented below in their separate sections.

Sigmoidal–Boltzmann model has successfully been used to determine the critical micelle concentration of surfactant [19], to study the maximum change in viscosity against time during cross linking process [20], and to estimate the temperature of maximum rate (transition state) during carbon–iodine bond cleavage from ethyl iodide on the Pd (III) surface [21]. The equation and percolation threshold based on this model have already been mentioned earlier. According to this model, the percolation thresholds of conductivity for EVA and NBR composites filled with different carbons have been shown in Figure 4b–d, respectively, and the same with other parameters are reported in Table S3. It is observed from both the figure and table that the percolation thresholds of NC, EC, NP, EP, NF, and EF composites are 0.140 ± 0.005, 0.143 ± 0.004, 0.038 ± 0.006, 0.039 ± 0.005, 0.041 ± 0.002, and 0.023 ± 0.007, respectively. It is seen that the values of percolation thresholds are higher for Conductex black filled composites compared to Printex black and SCF filled composites. The reason behind these different values of percolation threshold has been discussed in our previous publications [2,3,4,22]. The value of percolation threshold for this model can also be obtained without plotting the derivative curve. Since *x*_0_ indicates the percolation threshold in the equation, its *x*-axis value corresponding to *y*-axis value (*A*_1_ + *A*_2_)/2 will be the value of percolation threshold. Herein, since ∆*x* is the steepness of the curve around *Y*_50_[(*A*_1_ + *A*_2_)/2], which means halfway between the minimum and maximum of the response, it implies that conductivity is increased faster around the percolation threshold for the present composite system.

### 3.3. Sigmoidal–Dose Response Model

This Dose Response model has been used in medical field to analyze a relationship between the mean maximum viral reduction load and plasma inhibitory quotient or liver partition coefficient-corrected inhibitory quotient, to analyze bioassays in herbicide, toxicology, and pharmacology, and to evaluate the dose response of anticancer agents [23,24,25]. The equation based on this model can be given as:

(3) $$Y=A_{1}+\frac{A_{2}-A_{1}}{1+10^{\left(\log x_{0}-x\right)P}}$$

where *A*_1_ is the bottom value of electrical conductivity; *A*_2_ is the top value of electrical conductivity; *x* is the volume fraction of the filler loading; log *x*_0_ is the *x*-axis value halfway between bottom and top value of electrical conductivity; and *P* is the steepness of the curve around log *x*_0_. It is also called Hill slope or slope factor. For a standard dose response curve, the value of Hill slope is unity. If this value is greater than unity, then the curve is steeper, while, at less than unity, the curve is shallow. On differentiation of Equation (3), we have:

(4) $$\frac{dy}{dx}=\frac{P\ln10\left(A_{2}-A_{1}\right)10^{\left(\log x_{0}-x\right)P}}{{\left\{1+10^{\left(\log x_{0}-x\right)P}\right\}}^{2}}$$

**Figure 5:**
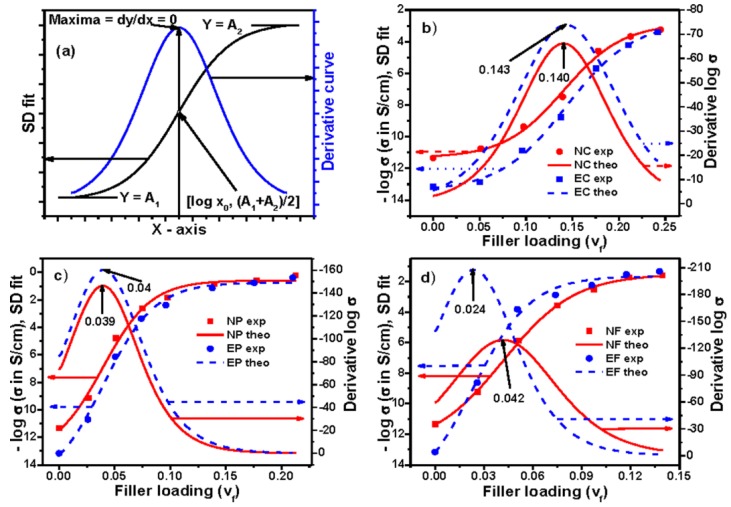
Experimental, theoretical, and derivative curves: (**a**) an ideal curve; (**b**) Conductex black filled; (**c**) Printex black filled; and (**d**) SCF filled composites based on Sigmoidal–Dose Response model.

It has been mentioned earlier that the first order differentiation gives the maximum of the equation and the magnitude of the maximum is obtained by equating the second order differentiation equal to zero; that is, *d*^2^*y*/*dx*^2^ = 0, which results *x* = log *x*_0_. Thus, log *x*_0_ indicates the percolation threshold in the above-mentioned equation. An ideal curve based on the above equation has been given in Figure 5a. The values of percolation thresholds, calculated from the derivative curves, are presented in Figure 5b–d. Table S4 shows the values of percolation thresholds along with other parameters. It is interesting to see that the values of percolation thresholds calculated using Sigmoidal–Boltzmann model and Sigmoidal–Dose Response model are the same. This is because, at percolation threshold, the value of *y*-axis for both models is equal; that is, (*A*_1_ + *A*_2_)/2. Hence, in this case, we can also get the value of percolation threshold directly from the plots by viewing the *x*-axis value at the corresponding *y*-axis (*A*_1_ + *A*_2_)/2. Herein, *P*, the steepness of the curve around *Y*_50_[(*A*_1_ + *A*_2_)/2], also implies that conductivity is increased faster around percolation threshold for the present composite system.

### 3.4. Sigmoidal–Hill Model

Levasseur and his coworkers applied this modified Hill model to find the time dependent behavior of in vitro drug cytotoxicity [26]. Later, this model has been extensively used in pharmacodynamic modeling for hematological toxicity for clinical practices and veterinary pharmacology, in an automated fitting procedure for multiphasic features, etc. [27,28,29,30]. The equation based on Sigmoidal–Hill model can be given as follows:

(5) $$Y=A_{1}+\frac{A_{2}-A_{1}}{1+{\left(k/x\right)}^{n}}$$

In another form, it can be written as:

(6) $$Y=A_{1}+\left(A_{2}-A_{1}\right)\frac{x^{n}}{k^{n}+x^{n}}$$

where *Y* is the dependent parameter of electrical conductivity at any volume fraction of independent parameter *x*; *A*_1_ and *A*_2_ are the initial and final values of electrical conductivity, respectively; *n* is the Hill coefficient, also known as hill slope/shape factor; and *k* is the volume fraction of filler at which *Y* = (*A*_1_ + *A*_2_)/2. On differentiation of the above equation, we have:

(7) $$\frac{dy}{dx}=\left(A_{2}-A_{1}\right)\frac{nk^{n}x^{n-1}}{{\left(k^{n}+x^{n}\right)}^{2}}$$

Hence, equating *d*^2^*y*/*dx*^2^ = 0, the percolation threshold value can be obtained from this model by making the same type of mathematical arguments that has been mentioned earlier, and is given as:

(8) $$x=k{\left(\frac{n-1}{n+1}\right)}^{\frac{1}{n}}$$

Thus, the percolation threshold depends on the magnitude of *n* (Hill coefficient > 1) and *k*, which in turns depend on the initial and final value of electrical properties. Putting the value of percolation threshold *x* in Equation (6), we have:

(9) $$Y=\frac{A_{1}k^{n}\left(n+1\right)+A_{2}k\left(n-1\right)}{k^{n}\left(n+1\right)+k\left(n-1\right)}$$

**Figure 6:**
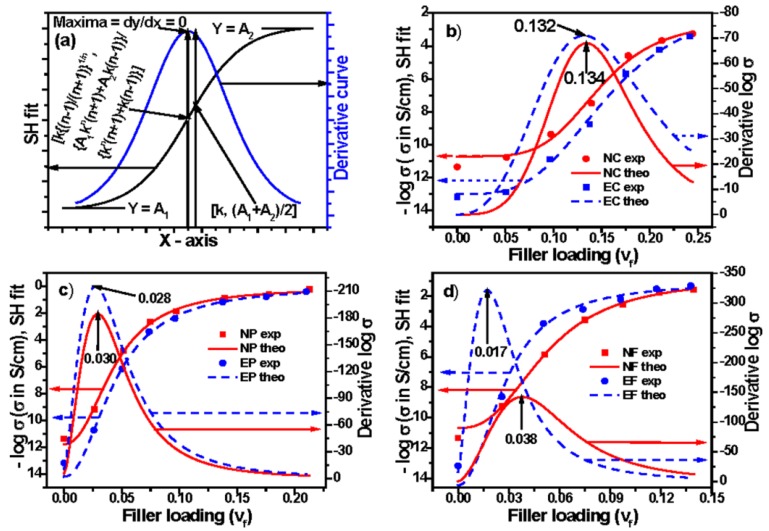
Experimental, theoretical, and derivative curves: (**a**) an ideal curve; (**b**) Conductex black filled; (**c**) Printex black filled; and (**d**) SCF filled composites based on Sigmoidal–Hill model.

This gives the value of *y*-axis parameter at the percolation threshold. Hence, an ideal plot based on this equation and subsequent derivations can be drawn, as shown in Figure 6a. The values of percolation thresholds, obtained from the maxima of the plots are shown in Figure 6b–d. Moreover, the values of parameters, obtained by fitting of the equation are presented in Table S5. This fitting gives the value of parameters *A*_1_, *A*_2_, *k*, *n*, and *R*^2^. Using parameters *k* and *n*, the value of percolation threshold *k*[(*n* − 1)/(*n* + 1)]^1/*n*^ has been calculated, which is also shown in Table S5. It is observed in both the figure and table that the values of percolation threshold resemble with each other. A careful look into the tables or figures reveals that the value of percolation threshold obtained through Sigmoidal–Hill model is always lower compared to Sigmoidal–Boltzmann and Sigmoidal–Dose Response models for any particular composite system. Actually, the value of *Y*_50_ (value of *Y* parameter at its 50%) for all these three models is the same: (*A*_1_ + *A*_2_)/2. The corresponding *x*-axis value for Sigmoidal–Boltzmann model is *x*_0_, for Sigmoidal–Dose Response model is log *x*_0_, and for Sigmoidal–Hill model it is *k*. The corresponding *x*-axis value of SB and SD models indicate their percolation threshold value, whereas, in the case of Hill model, it is not *k* but *k*[(*n* − 1)/(*n* + 1)]^1/*n*^, which indicates the percolation threshold value. The value of right hand term [(*n* − 1)/(*n* + 1)]^1/*n*^ is always less than unity. This is why we observe lower value of percolation threshold for Hill models compared to the two other models. For the present model, as *n* is the steepness of the curve around *Y*_50_[(*A*_1_ + *A*_2_)/2], it implies that conductivity is increased faster around *Y*_50_, but not around percolation threshold, because the percolation point is different from its *Y*_50_ level.

### 3.5. Sigmoidal–Logistic Model

This model is also known as Sigmoidal four-parameter logistic model, which has been used to describe the sigmoidal shaped response pattern for a long time [31,32]. It has been applied in the analysis of bioassays, radio-ligand assays, radio-immuno assays, and physiological assays [33,34]. The formula based on this four -parameter logistic model can be expressed as:

(10) $$Y=A_{1}+\frac{A_{2}-A_{1}}{1+{\left(x/x_{0}\right)}^{P}}$$

where *Y* is the response related to electrical conductivity at the filler loading *x*, *A*_1_ is the minimum/lower value of the conductivity, *A*_2_ is the maximum/upper value of the conductivity, *x*_0_ is the halfway between the minimum and maximum response and has the same unit as *x*, and *P* is the slope of the curve also known as slope factor (>1). The differentiation of the above equation gives the following result:

(11) $$\frac{dy}{dx}=\frac{\left(A_{2}-A_{1}\right)\left\{\frac{P}{x_{0}}{\left(x/x_{0}\right)}^{P-1}\right\}}{{\left[1+{\left(x/x_{0}\right)}^{P}\right]}^{2}}$$

Again, equating the second derivative (*d*^2^*y*/*dx*^2^) to zero, we have:

(12) $$x=x_{0}{\left(\frac{P-1}{P+1}\right)}^{\frac{1}{P}}$$

Hence, the parameter *x*_0_[(*P* − 1)/(*P* + 1)]^1/*P*^ give the value of percolation threshold for this equation. Putting this value into Equation (10), we can calculate its corresponding *y*-axis value:

(13) $$Y=\frac{A_{1}\left(P-1\right)+A_{2}\left(P+1\right)}{2P}$$

**Figure 7:**
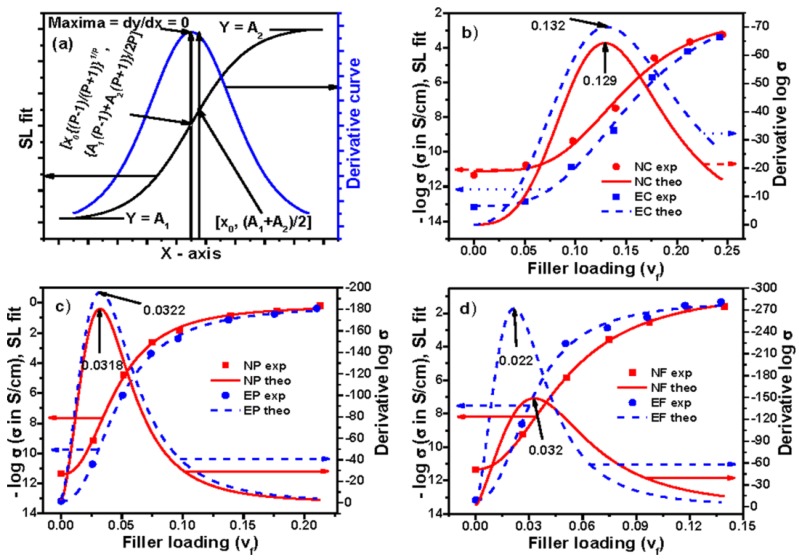
Experimental, theoretical, and derivative curves: (**a**) an ideal curve; (**b**) Conductex black filled; (**c**) Printex black filled; and (**d**) SCF filled composites based on Sigmoidal–Logistic model.

Thus, an ideal curve based on this equation and its subsequent derivatives can be plotted, which is shown in Figure 7a. To find the value of percolation threshold graphically, the experimental and derivative curves are also plotted in Figure 7b–d. The value of percolation thresholds, obtained from the maxima of the plots, has been mentioned within the figures. To calculate the value of percolation threshold, the data have been fitted with the equation and the obtained value of the parameters *A*_1_, *A*_2_, *x*_0_, and *P* are presented in Table S6 (see Supplementary Materials). Using the value of *x*_0_ and *P* in Equation (12), the value of percolation threshold has been calculated and also presented in Table S6. Interestingly, this calculated value of percolation threshold corroborates with the values obtained graphically. Again, a careful observation reveals that the value of percolation threshold obtained using the Logistic model is always lower compared to Boltzmann and Dose Response models. The reason behind this phenomenon is the same as in Hill model, and has been explained previously. Moreover, the value of percolation threshold obtained from Hill model and Logistic model are near but not equal. Although it appears that both models and percolation equations are the same, they are not exactly the same. The *x*-axis parameter and halfway *y*-axis parameter in both models are inversely related, and hence the corresponding *y*-axis value at the percolation threshold differs. For this SL model, the steepness of the curve, *P*, is around *Y*_50_[(*A*_1_ + *A*_2_)/2], hence this also implies conductivity increment is faster around *Y*_50_, but not around percolation threshold, because the percolation point is different from its *Y*_50_ level.

### 3.6. Sigmoidal–Logistic-1 Model

This three-parameter logistic model was first proposed by Pierre François Verhulst to study the population growth of human beings [35]. Singh et al. have utilized this equation in the growth modeling of *Lactococcas lactis* under various conditions [36]. There are also reports of the use of this model for wavelet analysis, bacterial growth analysis, etc. [37,38]. The equation based on this model can be given as:

(14) $$Y=\frac{A_{2}}{1+e^{-k\left(x-x_{c}\right)}}$$

where *Y* is the electrical conductivity at the corresponding filler loading *x*, *A*_2_ is the maximum value of electrical conductivity, *k* is the steepness of the curve, and *x*_c_ is the *x* value at the Sigmoidal midpoint. The differentiation of the above equation results as:

(15) $$\frac{dy}{dx}=\frac{kA_{2}e^{-k\left(x-x_{c}\right)}}{{\left\{1+e^{-k\left(x-x_{c}\right)}\right\}}^{2}}$$

**Figure 8:**
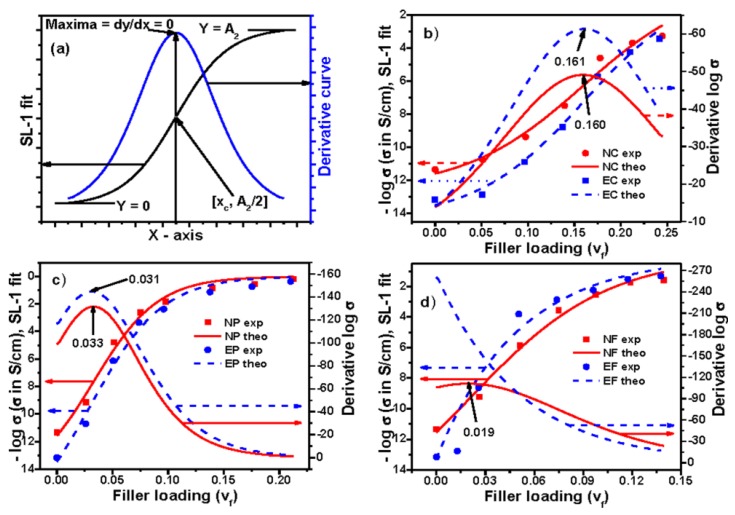
Experimental, theoretical, and derivative curves: (**a**) an ideal curve; (**b**) Conductex black filled; (**c**) Printex black filled; and (**d**) SCF filled composites based on Sigmoidal–Logistic-1 model.

Again, making *d*^2^*y*/*dx*^2^ = 0, we have *x* = *x*_c_. Hence, in the above-mentioned equation, *x*_c_ indicates the value of percolation threshold for this model. Putting *x* = *x*_c_ in the above equation, it results *Y* = *A*_2_/2. Hence, an ideal curve based on this model has been presented in Figure 8a. The experimental data and their derivative plots of electrical conductivity for all the composite systems are shown in Figure 8b–d. The values of percolation thresholds, obtained from the maximum of the curves, are mentioned with their respective plots. The values of percolation thresholds along with other parameters are reported in Table S7 (see Supplementary Materials). It is observed from the figure and table that the values of percolation thresholds for Conductex black field composites obtained from this model shows very high value compared to other models. Moreover, for NF and EF composites, it shows very low value and negative value, respectively. Actually, in this equation, the value of minima of the properties has not been considered during the proposition of this model, rather there is the value of maxima of the properties. However, in our data, for any particular composite system, there is the value of minima. Hence, this model will be more applicable to those systems where the values of minima will be equal to zero. 

### 3.7. Classical Percolation Theory and Model Comparison

Classical percolation theory was proposed by Kirkpatrick in 1973 [7]. This is also known as scaling law or power law behavior of percolation theory. Many authors have calculated the electrical percolation threshold value for their conductive polymer composite systems by applying this classical percolation theory in the past [37,38,39,40,41,42,43]. The equation based on this theory is given as:

(16) $$\sigma_{c}=\sigma_{0}{\left(V_{f}-V_{\mathrm{fc}}\right)}^{t},\;where,\;V_{f}>V_{\mathrm{fc}}$$

where *σ*_c_ is the conductivity of the composites, *V*_f_ is the volume fraction of fillers, *V*_fc_ is the volume fraction of fillers at the percolation threshold, *t* is the critical exponent, and *σ*_0_ is a constant quantity having the dimension of electrical conductivity. The value of critical exponent depends on the system dimension and has been given as between 1.65 and 2.0 for any three-dimensional network composite systems. Taking logarithm on both side of the above equation, we have:

(17) $$\log\sigma_{c}=\log\sigma_{0}+t*\log(V_{f}-V_{\mathrm{fc}})$$

**Figure 9:**
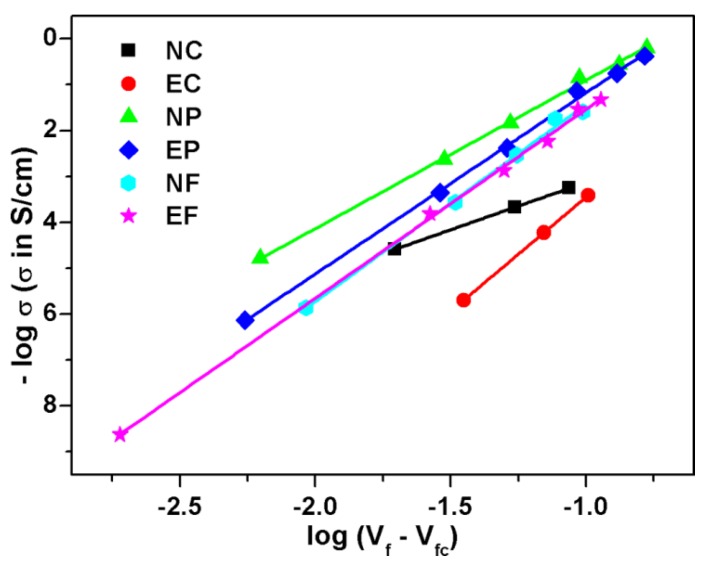
Plots of log *σ*_c_ vs. log (*V*_f_ − *V*_fc_) based on classical percolation theory for all the composite systems.

Thus, a plot of log *σ*_c_ vs. log (*V*_f_ − *V*_fc_) will give a straight line with the slope *t* and intercept log *σ*_0_. To determine the exact percolation threshold for our composite systems, we plotted log *σ*_c_ against log (*V*_f_ − *V*_fc_) and the value of *V*_fc_ was varied until the best linear fit was obtained. The plots of the best fit based on log *σ*_c_ vs. log (*V*_f_ − *V*_fc_) are shown in Figure 9, and the value of percolation thresholds along with critical exponent *t* and the coefficient of correlation (*R*^2^) are reported in Table S8 (see Supplementary Materials). It is seen in the table that the values of critical exponents are higher compared to its universal value mentioned earlier at the linear best fit. If one compares the percolation threshold value calculated from classical percolation theory with the other sigmoidal models (the percolation threshold values from all Sigmoidal models are summarized in Table S9, see Supplementary Materials), then it is revealed that the percolation threshold values calculated using classical theory are closer to the percolation threshold values estimated using Sigmoidal–Boltzmann and Sigmoidal–Dose Response models, except for NC composite. This discrepancy in the results may be due to lower value of critical exponent for NC composite compared to other systems (illustrated in Supplementary Materials (see critical exponent and percolation threshold section) by plotting a curve in Figure S2).

### 3.8. Applicability of Sigmoidal Models to Other Published Polymer Composite Systems

**Figure 10:**
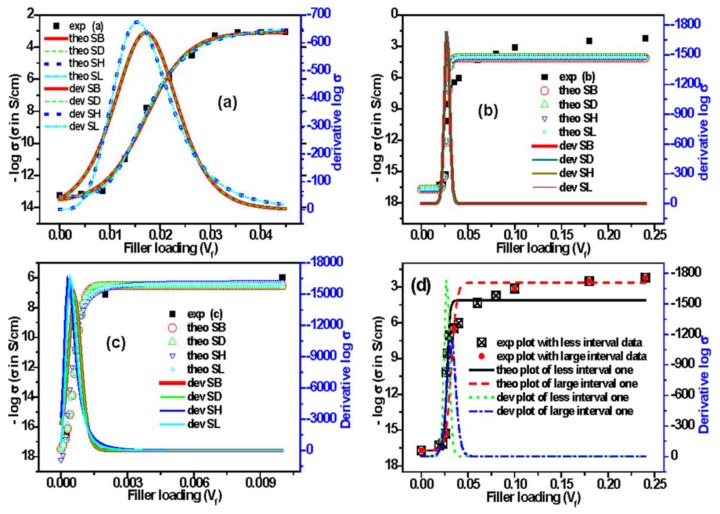
Experimental, different Sigmoidal and derivative curves: (**a**) Reference [44]; (**b**) Reference [45]; (**c**) Reference [46]; and (**d**) plots of Reference [45] based on less and large interval data points.

It is revealed from the above discussion that different Sigmoidal models can determine the percolation threshold value for our present composite systems. However, to check the applicability of these different Sigmoidal models for other composite systems, we have tested these models with the conductivity results in some published literature [44,45,46]. The filler loading and electrical conductivity in these studies have been converted into volume fraction and S/cm, respectively. The conductivity results from References [44,45,46] are for EVA/graphene composites, PP (polypropylene)/carbon black composites, and polyimides/single-walled carbon nanotubes (PI/SWCNTs) composites, respectively. The results from these studies, their Sigmoidal fitted curves, and derivative curves are presented in Figure 10a–c. The studies and model based percolation thresholds values are reported in Table S10. It is observed in Figure 10a that there are good fittings of Sigmoidal curves with the experimental results, although the value of percolation thresholds using different Sigmoidal models are a little higher compared to the literature value. On the contrary, the fitting curves based on different Sigmoidal models do not properly match the experimental results taken from Reference [45] (Figure 10b), but the percolation threshold values have exactly matched with each other. Figure 10c shows that the model based fitted curves of conductivity are mostly in good agreement with the conductivity results in Reference [46]. However, the percolation threshold value mentioned in this study, calculated by the classical percolation theory, is lower compared to Boltzmann and Dose response models, but is higher compared to Hill and Logistic models (Table S10, see Supplementary Materials). Thus, it can be said that the value of percolation threshold, calculated using the classical percolation theory, might be higher, equal or lower compared to the percolation thresholds values based on Sigmoidal models, depending on the nature of the experimental curve of conductivity. 

It has been mentioned earlier that the nature of Sigmoidal curve depends on the number of interval in the data points. To check the effect of number of interval data points on the percolation threshold value, the data from Reference [45] have been replotted in Figure 10d: one with the data having less interval data points, and the other with less number of data having large interval data points. Sigmoidal–Boltzmann model has only been applied to find the percolation threshold value for both cases, and its theoretical and derivative curves are also shown in Figure 10d. It is observed in the figure that the theoretical conductivity curve based on SB model has shifted to higher value when the intervals of the data points are large. Moreover, the derivative curves reveal that the value of percolation threshold has shifted from 0.027 to 0.032 when the intervals of the data points are large. Thus, it can be said that the number of data points affects the Sigmoidal conductivity, and the value of percolation threshold determined using it. In this case, we observed high value of percolation threshold when the intervals of data points are large for a particular composite system at the certain range of filler loading. 

## 4. Conclusions

The percolation threshold value from different types of Sigmoidal equations have been determined mathematically based on the concept of where the rate of increase in conductivity is maximum. Mathematically, the percolation threshold for SB, SD, SH, SL, and SL-1 models are *x*_0_, log *x*_0_, *k*[(*n* − 1)/(*n* + 1)]^1/*n*^, *x*_0_[(*P* − 1)/(*P* + 1)]^1/*P*^ and *x*_c_, respectively, where these are the corresponding *x*-axis value at the maximum of the derivative curve. It is observed that the percolation threshold value obtained from SB and SD models are similar. This is because the corresponding *y*-axis value at the percolation threshold for both equations are similar: (*A*_1_ + *A*_2_)/2. The percolation threshold values determined using SH and SL models do not coincide with each other and are of low value compared to SB and SD models. SL-1 model is not suitable for determining the percolation threshold value because a parameter, namely base line conductivity, is absent within the equation. The results of percolation threshold determined using Sigmoidal models are close enough to the results of percolation threshold calculated using the classical percolation theory. The physical validity of the fitting for these Sigmoidal models has been clearly understood from the value of coefficient of correlation (*R*^2^); that is, its closeness to unity. It is observed from the respective tables of these models that the value of *R*^2^ is close to unity for all models, except SL-1. The determination process of percolation threshold using these S-models is also easy: one only needs to fit the models. However, the percolation threshold value is affected by the number of interval in the data points: the higher is the number of data points, the lower is its percolation threshold for a particular composite system at their certain range of filler loading. These models have also been successfully used to determine the percolation threshold for other polymer conductive composite systems. Hence, it can be concluded that, except SL-1 model, the other sigmoidal models can universally be used to determine the percolation threshold value of electrical conductivity for extrinsically conductive polymer composite systems. It is hoped that researchers will also test these models when determining the percolation threshold value for their composite system, and then a strong base for these models will be established. 

## Figures and Tables

**Table 1:** Formulations of ethylene vinyl acetate (EVA) and acrylonitrile butadiene rubber (NBR) composites.

Ingredients	Composition Parts by Weight per Hundred Parts of Polymer		Composition by Volume Fraction (*V*_f_)	
	NBR Set	EVA Set	NBR Set	EVA Set
EVA	-----	100	-----	1-*V*_f_ of carbons
NBR	100	-----	1-*V*_f_ of carbons	-----
DCP	02	02	0.02	0.02
TAC	01	01	0.01	0.01
TQ	01	01	0.01	0.01
CCB	0–60	0–60	0–0.24477	0–0.24191
PCB	0–50	0–50	0–0.21265	0–0.21006
SCF	0–30	0–30	0–0.13945	0–0.13760

## Supplementary Materials

The following are available online at www.mdpi.com/2073-4360/9/10/527/s1, Figure S1: TEM image of (a) Structure of conductex black particle, (b) aggregated conductex black particle; FESEM image of (c) conductex black particle, (d) EC composite at low loading, and (e) EC composite at high loading; TEM image of (f) EC composite at high loading; TEM image of (g) a single printex black particle, (h) structure/aggregated printex black particle; FESEM image of (i) printex black particle, (j) EP composite at low loading, and (k) EP composite at high loading; TEM image of (l) EP composite at high loading; (m,n) are the optical microscopy of SCF; SEM image of (o) SCF, (p) EF composite of cryo-fractured sample, and (q) EF composite of solvent itched sample; TEM image of (r) EF composite showing a single fiber, Table S1: General specification of Conductex SC ultra beads and Printex XE2 carbon black; and so on.
