# Supplementary material for: A New Insight in Determining the Percolation Threshold of Electrical Conductivity for Extrinsically Conducting Polymer Composites through Different Sigmoidal Models
Source: Polymers (Basel). 2017 Oct 19;9(10):527. doi: 10.3390/polym9100527 (PMC6418723; doi:10.3390/polym9100527)
Supplement: Supplementary file 1 [file polymers-09-00527-s001.pdf]

polymers

MDPI

Supplementary Information

A new Insight in Determining the Percolation Threshold of Electrical Conductivity for Extrinsically Conducting Polymer Composites through Different Sigmoidal Models

Mostafizur Rahaman [1,*], Ali Aldalbahi [1,*], Periyasami Govindasami [1], Noorunnisa P. Khanam [2], Subhendu Bhandari [3], Peter Feng [4] and Tariq Altalhi [5]

1 Department of Chemistry, College of Science, King Saud University, Riyadh 11451, Saudi Arabia; pkandhan@ksu.edu.sa
2 Department of Mechanical and Industrial Engineering, Qatar University, Doha 2713, Qatar; noor.pathan@qu.edu.qa
3 Department of Plastics and Polymer Engineering, Maharashtra Institute of Technology, Aurangabad, Maharashtra 431010, India; subhenduonly@gmail.com
4 Department of Physics, University of Puerto Rico, San Juan, PR 00936-8377, USA; peter.feng@upr.edu
5 Department of Chemistry, Faculty of Science, Taif University, Taif 21974, Saudi Arabia; Ta.altalhi@tu.edu.sa
* Correspondence: mrahaman@ksu.edu.sa (M.R.); aaldalbahi@ksu.edu.sa (A.A.); Tel.: +966-114-675-883

C1: Calculation of Volume Fraction of Carbons

The volume fraction of carbons (V_f) with respect to polymers was calculated according to the formula $V_f = V_f/(V_p + V_f)$, where, V_p is the volume fraction of polymer. In this calculation process, the weighted mass (M) of carbons and polymers was converted into volume (V) by considering the density (D) of carbons, polymer EVA, and polymer NBR as 1.78 g/cm^3, 0.95 g/cm^3, and 0.98 g/cm^3, respectively as $V = M/D$.

Physical Characteristics of Different Carbon Fillers

The physical characteristics of conductex and printex carbon black are shown in Table S1. It is seen that the particle size, surface area, and DBP adsorption value of conductex carbon black is less compared to printex black. However, it can be said herein that the most important property of carbon black that governs the electrical conductivity of polymer composites is its structure (carbon particles are fused together to form a structure). The DBP absorption value gives an idea about the structure of carbon black. High value of DBP absorption indicates higher structure of carbon black. A high structure carbon black can form conductive continuous network within the polymer matrix at less volume fraction of filler. As a result, less value of percolation threshold is observed. This is why, printex black filled composites exhibit lower value of electrical percolation threshold compared to conductex black filled composites.

The percent volatile content gives an idea about the oxygenated content on the surface of carbons. It is seen from the table that the volatile content for both carbons are very less though conductex

black has higher value compared to printex black one. The present of volatile content reduce the electrical conductivity of carbons but to a very lesser extent. For the present composite systems, the overall variation in electrical conductivity is in the order of 10^{12} S/cm. Consequently, the effect of volatile content on electrical conductivity for the present composite systems is totally negligible, and hence cannot be accounted for.

Table S1. General specification of Conductex SC ultra beads and Printex XE2 carbon black.

Typical properties	Conductex	Printex	Unit
Mean particle size	20	35	nm
Surface area, STSA	125	587	m^2/g
Surface area, CTAB	130	600	m^2/g
DBP absorption	115	350–410	cc/100 g
Volatiles at 105 °C	1.5	1.0	%

The average length and aspect ratio of carbon fiber in different composites are presented in Table S2. The average length of carbon inside the polymer composites was measured using the optical microscopy imaging of the composites [Polymer Composites 32(11), 1790-1805, 2011]. The diameter of the fiber was 6.0 micron.

Table S2. Average length (AL) and aspect ratio (AR) of fiber within the composite materials.

Filler loading	NF		EF	
(wt%)	AL (mm)	AR	AL (mm)	AR
5	0.233	34.3	0.352	51.8
10	0.192	28.2	0.315	46.3
15	0.157	23.1	0.281	41.3
20	0.125	18.4	0.243	35.7
25	0.092	13.5	0.214	31.5
30	0.058	8.5	0.188	27.6

Table S3. Parameters based on Sigmoidal-Boltzmann model.

SI	A_1	A_2	x_0	dx	R^2

NC	11.32±0.30	2.85±0.40	0.140±0.005	0.032±0.005	0.992
EC	13.43±0.31	2.72±0.48	0.143±0.004	0.036±0.004	0.995
NP	13.26±1.32	0.57±0.25	0.038±0.006	0.022±0.003	0.989
EP	15.54±1.38	0.73±0.26	0.039±0.005	0.023±0.004	0.992
NF	12.85±0.51	1.50±0.16	0.041±0.002	0.022±0.002	0.998
EF	16.20±2.47	1.64±0.32	0.023±0.007	0.018±0.004	0.988

Table S4. Parameters based on Sigmoidal Dose Response model.

**SI**	**A_1**	**A_2**	**log x_0**	**p**	**x_0**	**R^2**
NC	2.85±0.40	11.32±0.30	0.140±0.005	-13.58±2.26	1.381	0.992
EC	2.72±0.48	13.43±0.31	0.143±0.004	-11.97±1.60	1.391	0.995
NP	0.57±0.25	13.26±1.32	0.039±0.006	-20.05±3.63	1.094	0.989
EP	0.73±0.26	15.54±1.38	0.040±0.005	-18.81±2.93	1.096	0.992
NF	1.50±0.16	12.85±0.52	0.042±0.002	-19.80±1.83	1.101	0.998
EF	1.64±0.32	16.20±2.47	0.024±0.007	-24.77±6.18	1.056	0.988

Table S5. Parameters based on Sigmoidal-Hill model.

**SI**	**A_1**	**A_2**	**k**	**n**	**Xp**	**R^2**
NC	10.70±0.55	02.40±1.14	0.1478±0.0120	4.6257±1.6372	0.134	0.978
EC	13.01±0.46	01.13±1.59	0.1567±0.0133	3.4456±0.7969	0.132	0.993
NP	11.75±2.11	0.062±0.39	0.0439±0.0077	2.2803±0.5967	0.030	0.996
EP	14.19±4.59	0.036±0.95	0.0441±0.0142	2.1219±0.9958	0.028	0.988
NF	10.68±1.99	0.814±1.17	0.0521±0.0075	2.5793±1.2843	0.038	0.989
EF	14.45±6.94	1.059±2.70	0.0278±0.0897	2.1237±5.0932	0.017	0.966

Table S6. Parameters based on Sigmoidal-Logistic model.

**SI**	**A_1**	**A_2**	**x_0**	**P**	**Xp**	**R^2**
NC	11.09±0.32	1.95±1.18	0.147±0.012	3.89±1.01	0.129	0.985

EC	13.12±0.26	0.85±1.53	0.158±0.014	3.29±0.60	0.132	0.993
NP	11.38±0.16	0.14±0.15	0.045±0.003	2.46±0.14	0.0318	0.998
EP	13.19±0.14	0.14±0.14	0.047±0.001	2.36±0.10	0.0322	0.999
NF	11.33±0.11	0.51±0.22	0.050±0.002	2.24±0.11	0.032	0.999
EF	13.18±0.33	1.18±0.37	0.031±0.002	2.38±0.33	0.022	0.994

Table S7. Parameters based on Sigmoidal-Logistic-1 model.

**SI**	**A_2**	**x_c**	**k**	**R^2**
NC	12.53±1.04	0.160±0.014	-15.6±2.8	0.972
EC	14.37±0.78	0.161±0.009	-17.1±2.2	0.985
NP	15.28±2.84	0.033±0.013	-34.4±6.8	0.984
EP	18.48±3.64	0.031±0.014	-31.3±5.9	0.985
NF	18.87±5.32	0.019±0.025	-23.5±4.2	0.984
EF	47.47±77.20	-0.036±0.104	-24.2±8.1	0.963

Table S8. Parameters based on classical percolation theory.

**SI**	**V_{fc}**	**Slope**	**R^2**
NC	0.158	2.06934	0.999
EC	0.140	4.971	1.000
NP	0.045	3.2305	0.998
EP	0.045	3.9449	0.998
NF	0.042	4.297	0.995
EF	0.024	4.10986	0.998

Table S9. Percolation threshold value of all Sigmoidal models.

**SI**	**Percolation threshold values**				
	**SB**	**SD**	**SH**	**SL**	**SL-1**
NC	0.140±0.005	0.140±0.005	0.134	0.129	0.160±0.014
EC	0.143±0.004	0.143±0.004	0.132	0.132	0.161±0.009

NP	0.038±0.006	0.039±0.006	0.030	0.0318	0.033±0.013
EP	0.039±0.005	0.040±0.005	0.028	0.0322	0.031±0.014
NF	0.041±0.002	0.042±0.002	0.038	0.032	0.019±0.025
EF	0.023±0.007	0.024±0.007	0.017	0.022	-0.036±0.104

Table S10. Percolation thresholds value for references 45-47 (EP) and their fittings based on different Sigmoidal models.

**SI**	**Percolation threshold values**				
	**SB**	**SD**	**SH**	**SL**	**EP**
**R45**	0.017	0.017	0.015	0.015	0.013
**R46**	0.027	0.027	0.027	0.027	0.027
**R47**	0.0006	0.0006	0.0003	0.0004	0.0005

Morphological Study

Characterizations

The morphological analysis of carbon blacks and their composites has been carried out through transmission electron microscopy (TEM), scanning electron microscopy (SEM), field emission scanning electron microscopy (FESEM), and optical microscopy.

The instrument used to study the morphology of carbons and their composites through SEM analysis was JEOL JSM 5800 scanning electron microscope (Tokyo, Japan). All samples were gold coated prior to analysis using vacuum gold-sputter machine. The SEM study has carried out on cryo-fractured surface of vulcanized samples and etched surface of unvulcanized samples.

The distribution and morphology of carbons into the polymer matrix were studied using a high resolution transmission electron microscope (HRTEM, JEM 2100, JEOL Limited, Tokyo, Japan) attached with charge couple device (CCD) camera (Gatan, Inc., CA, USA). The specifications for HRTEM were point to point resolution of 0.194 nm, lattice resolution of 0.14 nm, 24° tilt angle, accelerated voltage of 200 keV and electron gun of lanthanum hexaboride (LaB6) filament type. The samples for HRTEM analysis were prepared using an ultramicrotomy with a Leica Ultracut UCT (Leica Microsystems GmbH, Vienna, Austria). Freshly sharpened diamond knives with cutting edges of 45° were used to obtain cryosections of 50–60 nm thickness specimens at ambient temperature. The cut samples were supported on a copper mesh before observation under the microscope. To study the shape and size of carbon particle, the particle were

dispersed in acetone for one hour using bath type sonicator and then it was placed on copper mesh to perform the morphological study.

The morphology of carbons and polymer/carbon composites was evaluated using field emission scanning electron microscope (model Supra 40, Carl Zeiss SMT AG, Oberkochen, Germany). Samples were gold coated by means of manually operated sputter coater (model SC7620, Polaron Brand, Quorum Technologies Ltd., East Sussex, UK) unit.

For calculating fiber average length (already reported in the above section) and their distribution in composite materials, optical microscopy was used. To perform this test, carbon fibers were extracted from unvulcanized samples of different composites through solvent extraction method.

Morphology of Carbons and their Composites

The morphological analysis of carbon blacks and their composites, carried out through TEM, SEM) FESEM) and optical microscopy, are shown in Fig. S1. The morphology of conductex black and its composites is shown in Fig. S1 (a-f), printex black and its composites is shown in Fig. S1 (g-l), and SCF and its composites is shown in Fig. S1 (m-r). It is seen from the TEM images that the carbon black particles are existed as aggregated form called its structure (Fig. S1 a and h). The particle size/structure of printex carbon black is much higher than that of conductex black. This is one of the reason of getting higher conductivity and low percolation threshold of printex black filled composites compared to conductex black filled one. The carbon fibers are long sticks and are having high length to diameter ratio as is observed from optical microscopy, SEM, and TEM images. Hence, we get same type of observation that is higher electrical conductivity and low percolation threshold for carbon fiber filled composites compared to conductex filled composite one. FESEM images also show that the particle size of printex black is higher compared to conductex black within the composites. At low filler loading, the particles aggregates are isolated from each other (Fig. S1 d and j) but for higher loaded composite, the aggregates are compact with each other and form conductive continuous network (Fig. S1 e and k). Similar thing is observed for carbon fiber filled composites. The cryo-fractured surface of carbon fiber composite shows breakage of fibers (Fig. S1 p); whereas, surface itched sample of carbon fiber composites shows interconnected fiber network within the polymer matrix (Fig. S1 q). This interconnected network helps in the conduction of charge carriers throughout the composite system and hence results in higher conductivity. The fibers are having very low bending strength. As a result, there is the breakage of it during cryo-fracture process. Also, it is seen that there is the holes within the polymer matrix which has

happened due to pulling out of the fibers. This indicates low fiber-polymer interaction within the composite.

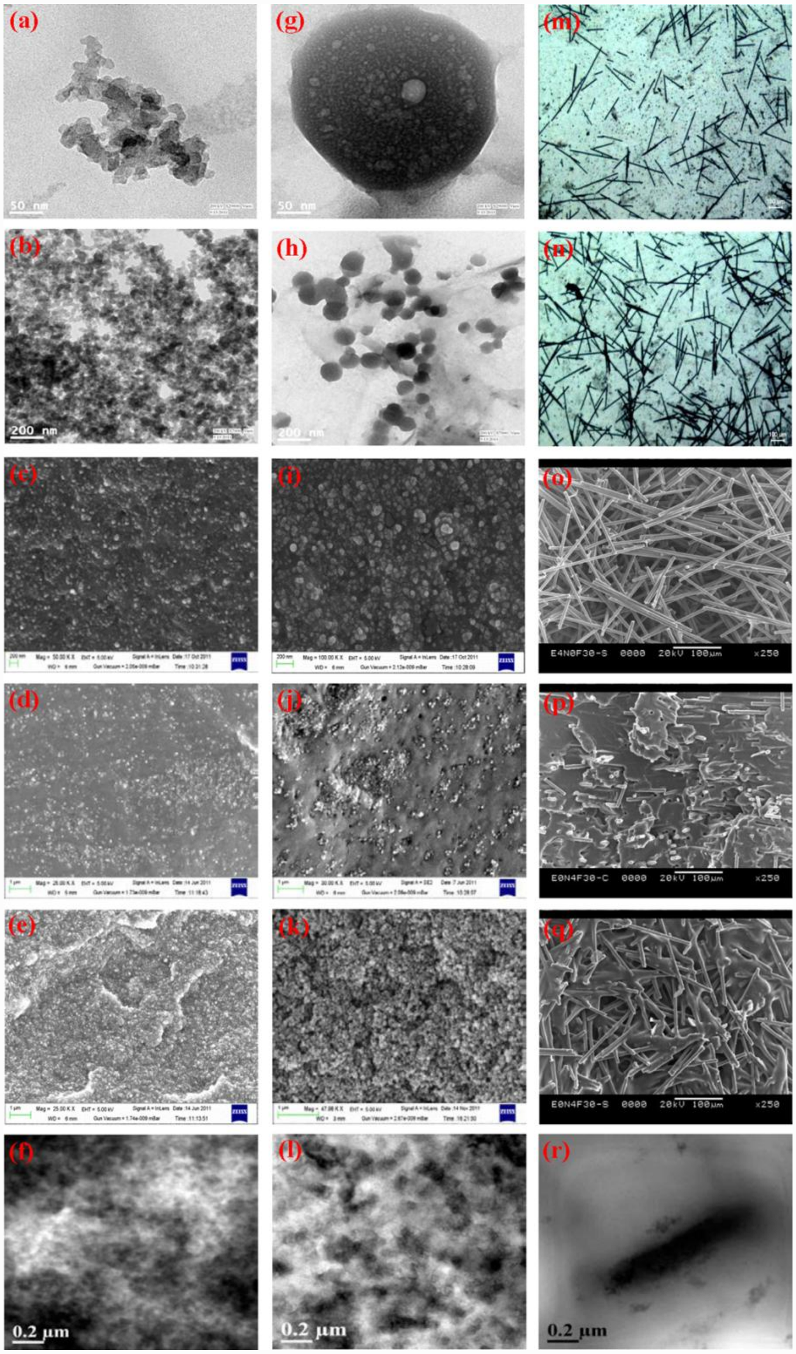

Figure S1. TEM image of (a) Structure of conductex black particle, (b) aggregated conductex black particle; FESEM image of (c) conductex black particle, (d) EC composite at low loading, and (e) EC composite at high loading; TEM image of (f) EC composite at high loading; TEM image of (g) a single printex black particle, (h) structure/aggregated printex black particle; FESEM image of (i) printex black particle, (j) EP composite at low loading, and (k) EP composite at high loading; TEM image of (l) EP composite at high loading; (m) and (n) are the optical microscopy of SCF; SEM image of (o) SCF, (p) EF

composite of cryo-fractured sample, and (q) EF composite of solvent itched sample; TEM image of (r) EF composite showing a single fiber.

Critical Exponent (slope value) and Percolation Threshold

The value of critical exponent *t* in the classical percolation theory is determined from the slope of the linear plot of $log\ \sigma$ vs $log\ (V_f - V_{fc})$ as mentioned earlier within the manuscript. Actually, this linear plot [plotting $log\ \sigma$ vs $log\ (V_f - V_{fc})$] corresponds to the exponential nature of conductivity curve [when plotted $log\ \sigma$ vs V_f] at and beyond percolation threshold. Hence, a correlation curve is shown in illustrative Fig. S2. In this figure two slopes with different value (black line less value; whereas, red line high value) are plotted with their corresponding percolation threshold value. It is seen that with the lowering of slope value, the percolation point has shifted to higher value and hence exhibited higher percolation threshold value.

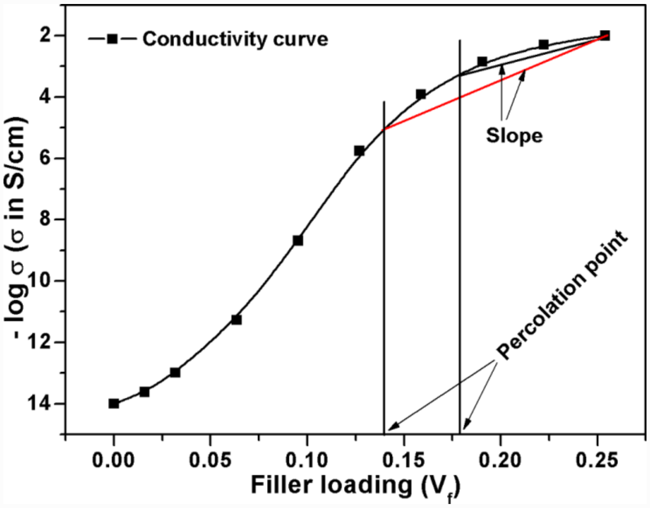

Figure S2. Correlation of critical exponent (slope value) with percolation threshold.
